# CO_2_ conversion to formamide using a fluoride catalyst and metallic silicon as a reducing agent

**DOI:** 10.1038/s42004-022-00767-4

**Published:** 2022-11-16

**Authors:** Ruopeng Wang, Kaiki Nakao, Yuichi Manaka, Ken Motokura

**Affiliations:** 1 Department of Chemistry and Life Sciences, Yokohama National University, 79-5 Tokiwadai, Hodogaya-ku, Yokohama, 240-8501 Japan; 2 Department of Chemical Science and Engineering, Tokyo Institute of Technology, 4259 Nagatsuta-cho, Midori-ku, Yokohama, 226-8502 Japan; 3 Renewable Energy Research Center, National Institute of Advanced Industrial Science and Technology (AIST), 2-2-9 Machiikedai, Koriyama, 963-0298 Japan

**Keywords:** Heterogeneous catalysis, Sustainability, Materials for energy and catalysis

## Abstract

Metallic silicon could be an inexpensive, alternative reducing agent for CO_2_ functionalization compared to conventionally used hydrogen or hydrosilanes. Here, metallic silicon recovered from solar panel production is used as a reducing agent for formamide synthesis. Various amines are converted to their corresponding amides with CO_2_ and H_2_O via an Si-H intermediate species in the presence of a catalytic amount of tetrabutylammonium fluoride. The reaction system exhibits a wide substrate scope for formamide synthesis. Spectroscopic analysis, including in situ Fourier transform infrared (FTIR), X-ray photoelectron spectroscopy (XPS), X-ray diffraction (XRD), N_2_ adsorption/desorption analyses, and isotopic experiments reveal that the fluoride catalyst effectively oxidizes Si atoms on both surface and interior of the powdered silicon particles. The solid recovered after catalysis contained mesopores with a high surface area. This unique behavior of the fluoride catalyst in the presence of metallic silicon may be extendable to other reductive reactions, including those with complex substrates. Therefore, this study presents a potential strategy for the efficient utilization of abundant resources.

## Abstract (web summary)

The reductive functionalization of CO_2_ to value-added chemicals using powdered silicon as a reducing agent is a promising sustainable route, but the behavior of employed fluoride catalysts remains largely unknown. Here, the authors use tetrabutylammonium fluoride to catalyze the conversion of a variety of amines to amides with CO_2_ and H_2_O reduced by metallic silicon recovered from solar panel production waste and show that the catalyst effectively oxidizes Si atoms on both the surface and interior of the powdered silicon particles.

## Introduction

Carbon dioxide emissions have been increasing since the Industrial Revolution^1^. Considering this situation, the United Nations Environment Programme (UNEP) predicted that a 7.6% annual reduction of CO_2_ emissions for at least a decade is necessary to limit the global temperature increase in 2100 to 1.5 °C above the pre-industrial level^2^. The UNEP agency suggested prioritizing direct support for zero-emission technologies and infrastructure, reducing fossil fuel subsidies, ceasing new coal plants, and promoting nature-based solutions.

With increasing environmental and social concerns regarding carbon emissions, the utilization and conversion of CO_2_ as a sustainable C1 building block has attracted extensive interest because CO_2_ is an abundant, inexpensive, nontoxic, and renewable C1 source^3–6^. However, owing to its thermodynamic stability and kinetic inertness, this task is energetically intensive and requires highly reactive nucleophiles and/or transition-metal catalysts for CO_2_ activation. While the majority of studies have focused on metal-catalyzed reactions and functionalization of CO_2_, organocatalysis for CO_2_ conversion reactions is also a highly active research area. Recently, *N*-heterocyclic carbenes (NHCs)^7^, 1,5,7-triazabicyslo[7.4.0]dec-5-ene (TBD)^8^, thiazolium carbene^9^, 1,3,2-diazaphosphatrane (NHP-H)^10^, carbodicarbenes (CDCs)^11^, tetrabutylammonium formate^12^, and others^13–19^ have been reported to exhibit high catalytic activity comparable with metal-based catalysts. The use of a cheap reducing agent, polymethylhydrosilane, was also investigated using an organocatalyst^20^. The CO_2_ conversion reaction using organocatalysts provides an opportunity for new carbon capture, utilization and storage (CCUS) technologies^21–25^.

The thermodynamic stability of CO_2_ is derived from its strong C-O bonds; therefore, reductants are required for reductive CO_2_ functionalization. H_2_ and hydrosilanes are often selected as reductants. The reduction of CO_2_ by hydrosilanes is an exothermic reaction, affording silyl formate as a product. However, relatively expensive hydrosilanes are consumed stoichiometrically, which is not economically feasible.

**Fig. 1:**
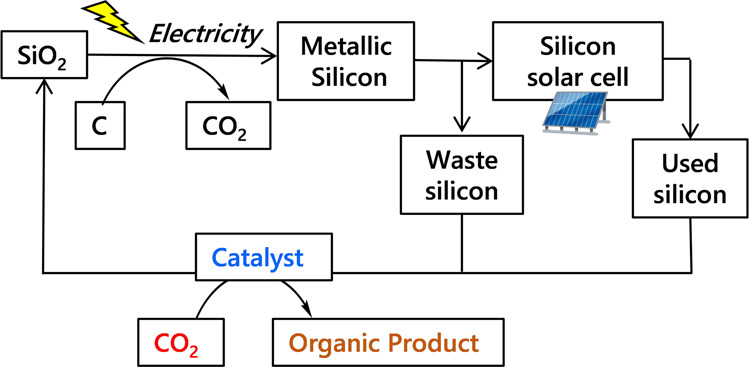
Proposed Si recycling process with CO_2_ conversion to organic products. Waste/used silicon could be recycleed as a reducing agent of CO_2_ to form value-added organic products.

**Fig. 2:**
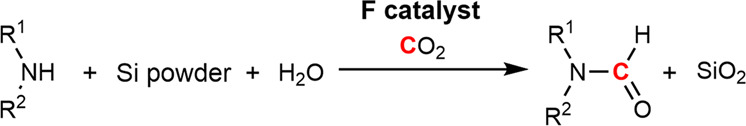
Reductive CO_2_ functionalization using waste Si and amines. Fluoride catalysis for this reaction was investigated.

Metallic Si is an inexpensive alternative reducing agent. The International Renewable Energy Agency (IRENA) estimated that in 2050, there will be 60–78 MT of global PV panel waste, of which two-thirds is of the crystalline silicon variety^26^. The current economic value of these panels is low, despite the high purity of silicon wafers^27, 28^. Therefore, utilizing waste Si from the production process of solar panels to reduce CO_2_ to organic chemicals and energetic compounds generates a circular economy that is beneficial to the environment. This is a novel concept in the Si recycling process, as shown in Fig. 1. Our group and others have previously reported the reduction of CO_2_ to formic acid and methanol using powdered silicon as a reducing agent^29–34^. However, the detailed fluoride catalysis for not only reductive functionaization of CO_2_ for the application of chemical synthesis but also the behavior of fluoride at both surface and interior of Si powder are still unknown. In this study, in order to greatly expand the fluoride catalysis on reductive conversion of CO_2_, the successful synthesis of formamides in high yields using CO_2_ as a C1 carbon source and silicon wafers recovered from the solar panel production process as a reducing agent is reported (Fig. 2). The reaction successfully proceeded in the presence of a fluoride catalyst. Spectroscopic, kinetic, and isotopic experiments, including in situ measurements, revealed the reaction mechanism, including both surface and interior events on the metallic silicon reductant. This is the first report to present highly efficient formamide synthesis from CO_2_ using metallic silicon as a reducing agent with unique catalysis of fluoride with metallic silicon.

## Results and discussion

### Formamide synthesis from amine and CO_2_ with Si powder

The reductive transformation of CO_2_ with morpholine and metallic silicon was investigated using various fluoride catalysts, as shown in Table 1. The reaction proceeded well with fluoride salts with large cation sizes, such as tetrabutylammonium fluoride (TBAF) and tetraethylammonium fluoride (TEAF), and afforded *N*-formylmorpholine. When TBAF catalyst (0.05 mmol) was used, the amount of product (1.91 mmol) was approximately 40 times larger than the amount of catalyst, suggesting that the catalytic cycle was active. The yield based on CO_2_ used is calculated to be *ca*. 18%. In contrast, inorganic salts with relatively smaller cation sizes^35, 36^ and lower solubility^37^ in organic solvent, such as CsF, KF, and NaF, showed lower reactivity. These results suggest that the activity of the catalyst is related to the counter cation size and solubility in fluoride salts. When other halogens, such as Cl, Br, and I, were used as counter anions, the reaction barely proceeded. This is thought to be owing to the fact that the Si-F bond (e.g. Me_3_Si-F: 158 kcal mol^−1^)^38^ has a much higher dissociation energy than other bonds such as Si-Si and Si-Cl (e.g. Me_3_Si-SiMe_3_: 79.3 kcal mol^−1^; Me_3_Si-Cl: 117 kcal mol^−1^)^39^, and thereby fluoride facilitates the Si-Si bond cleavage.

**Table 1:** CO_2_ conversion to formamide with various fluoride catalyst^a^.


Catalyst	Yield (mmol)^b^
TBAF	1.91
TBAF(t-BuOH)_4_	1.42
TEAF	1.15
CsF	<0.01
KF	0.10
NaF	0.09
TBACl	<0.01
TBABr	<0.01
TBAI	<0.01
None	<0.01

^a^Reaction conditions: Si powder (5.0 mmol), morpholine (3.0 mmol), NMP (4 mL), H_2_O (10 mmol), TBAF (0.05 mmol), CO_2_ (9 atm), 90 °C, 24 h.

^b^Determined by GC-FID using an internal standard technique.

Table 2 summarizes the results of the catalytic reactions under various reaction conditions. The formylated product was obtained in 94% yield at 6 atm and 120 °C (Entry 1). The CO_2_ pressure and reaction temperature were decreased to 4 atm and 90 °C, respectively, while maintaining a high yield (Entry 2), indicating high catalytic performance of TBAF. The product was not obtained without H_2_O or CO_2_ (Entries 5 and 6). These results suggest that both H_2_O and CO_2_ are converted to formamide product possibly. The use of excess amount of powdered silicon enables effective production of the formamide.

**Table 2:** CO_2_ conversion to formamide with fluoride catalyst^a^.


Entry	H_2_O (mmol)	TBAF (mmol)	CO_2_ (atm)	Yield (%)^b^
1	10	0.05	6	94
2	10	0.05	4	87 (90 °C)
4	10	None	6	<1
5	None	0.05	6	<1
6	10	0.05	None^c^	N.D.^d^
7	10	None	None^c^	N.D.^d^

^a^Reaction conditions: Si powder (5.0 mmol), morpholine (1.0 mmol), DMSO (4 mL), H_2_O (10 mmol), TBAF (0.05 mmol), CO_2_ (6-4 atm), 120 °C, 72 h.

^b^Determined by GC-FID using an internal standard technique.

^c^Under Ar (6 atm).

^d^N.D. = not detected.

The effects of different solvents are shown in Table 3. The reaction proceeded well in aprotic polar solvents with C = O and S = O double bonds, such as DMSO, DMA, NMP, and DMF. Further optimization of reaction conditions enabled a quantitative yield of the corresponding formamide product (>99%). In contrast, and surprisingly, the reaction did not proceed well in CH_3_CN compared with NMP, despite its polarity. This suggests that the electron donation of solvents with C = O or S = O bonds to Si-H on the silicon wafer powder during the reaction increases the electron density of Si, which facilitates the reaction of CO_2_^12^. Weakly polar solvents, such as THF, dioxane, and CH_3_Cl, were unsuitable for this reaction. Furthermore, the reaction barely proceeded in nonpolar solvents such as *n*-hexane and toluene.

**Table 3:** Solvent effect on fluoride-catalyzed CO_2_ conversion to formamide^a^.


Solvent	Dielectric constant (ε)	Yield of amide (%)^b^
DMA	37.8	>99
DMF	38	>99
NMP	32	>99
DMSO	47.2	95
THF	7.5	22
dioxane	2.2	13
MeCN	37.5	12
*n*-hexane	1.9	2
CH_3_Cl	4.8	2
toluene	2.4	1
H_2_O	80	<1

^a^Reaction conditions: Si powder (5.0 mmol), morpholine (1.0 mmol), solvent (4 mL), H_2_O (10 mmol), TBAF (0.05 mmol), CO_2_ (9 atm), 90 °C, 24 h.

^b^Determined by ^1^H NMR spectroscopy using an internal standard technique.

Based on the optimized reaction conditions, the scope of this formylation was further investigated using various amines. Table 4 shows the scope of amines in the formylation reaction at 9 atm of CO_2_ using powdered waste Si and TBAF as the reducing agent and catalyst, respectively. Various cyclic and linear aliphatic secondary amines showed high reactivity, affording the corresponding amides in 72–99% yields (Entries 1–5). *N*-Methylbenzylamine also showed good reactivity and afforded a high yield of amide (Entry 6). A primary amine, benzylamine, also underwent formylation and 85% of the corresponding amide was obtained with a trace amount of the diformylated product. Weakly nucleophilic aromatic amines were unsuitable for this reaction (Entry 8).

**Table 4:** Substrate scope of amine on CO_2_ conversion to formamide^a^.

^a^Reaction conditions: Si powder (5.0 mmol), morpholine (1.0 mmol), NMP (4 mL), H_2_O (5 mmol), TBAF (0.05 mmol), CO_2_ (9 atm), 120 °C, 72 h.

^b^Determined by ^1^H NMR using an internal standard technique.

^c^90 °C, 24 h.

### Mechanistic investigation

**Fig. 3:**
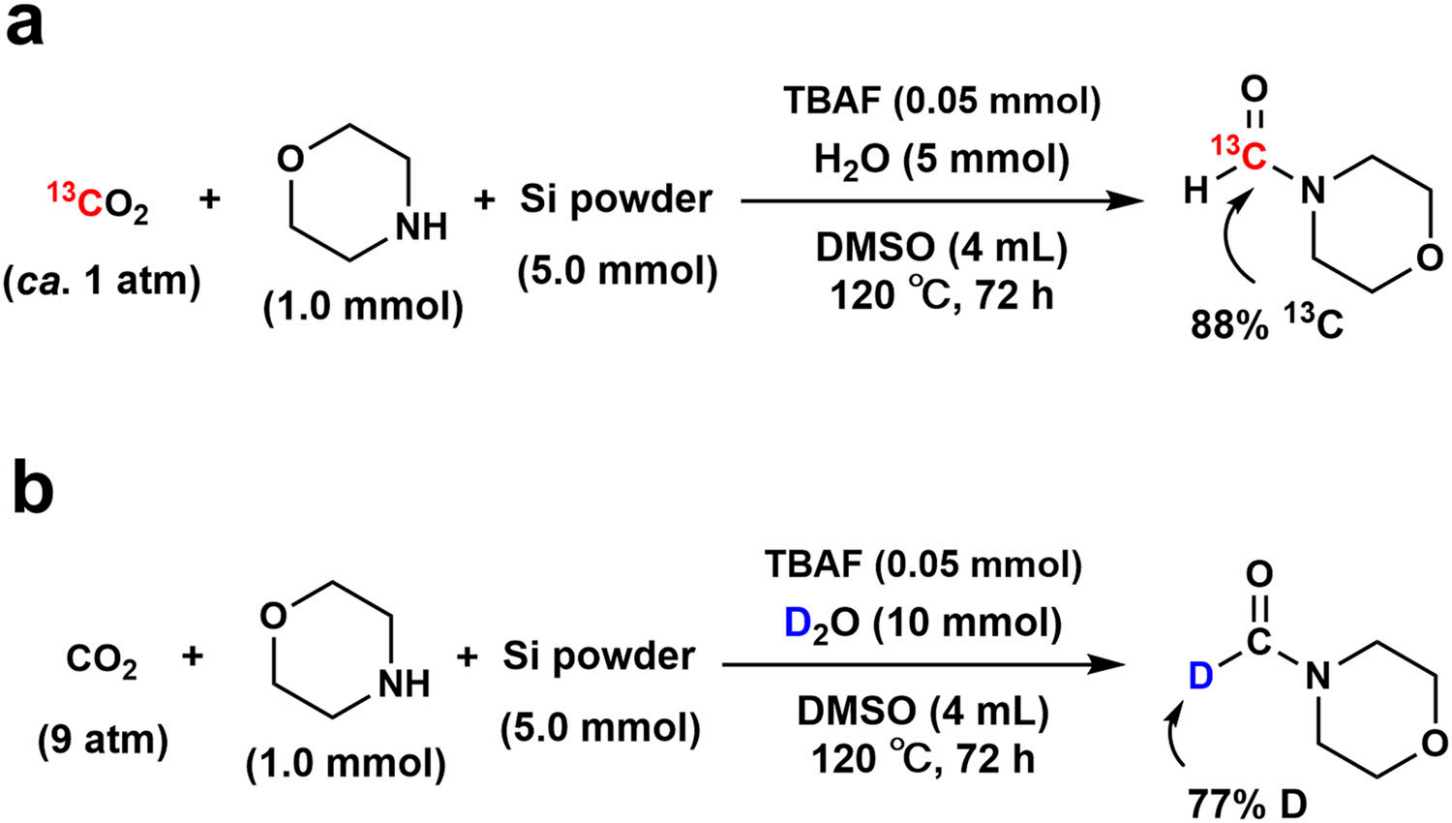
Isotopic experiment of fluoride-catalyzed CO_2_ conversion to formamide. The reaction using (**a**) ^13^CO_2_ and (**b**) D_2_O instead of ^12^CO_2_ and H_2_O, respectively.

To confirm formamide formation was derived from CO_2_, ^13^CO_2_ (99 atom%^13^C) was used. ^1^H NMR measurements confirmed that the peaks assigned to the formyl group (8.1 ppm) were split by ^13^C-^1^H coupling (*J* = 193.5 Hz) (see Supplementary Fig. S1). ^13^C NMR results showed that the peak originating from the formyl group was much larger in the reaction that used ^13^CO_2_ than in the reaction with ^12^CO_2_. Finally, in GC–MS fragmentation spectra, it was observed that the maximum value of the molecular ion peak was 115 for the reaction with normal ^12^CO_2_, whereas the maximum value increased to 116 for the reaction with ^13^CO_2_. These results suggest that the carbon atom of CO_2_ was introduced into the amide formyl group (Fig. 3). The proton source for the formylation reaction was also evaluated by applying deuterium oxide as the proton source instead of H_2_O. It was confirmed that the maximum molecular ion peak value in the fragment GC–MS spectra of the amide was 115 in the case of using ordinary H_2_O, whereas the maximum value shifted to 116 in the case of using D_2_O (see Supplementary Fig. S2). These results indicate that the hydrogen atom of the formyl group in the formamide product was derived from H_2_O (Fig. 3).

**Fig. 4:**
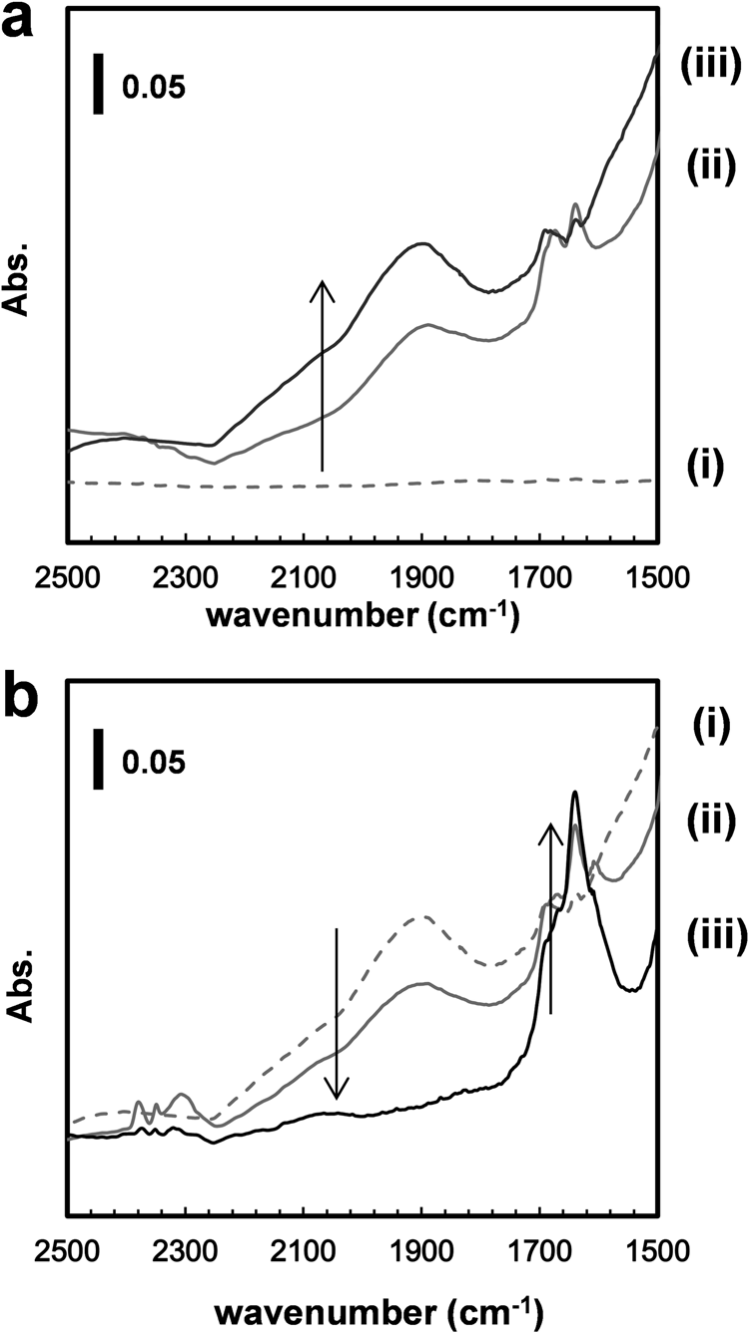
In situ FTIR spectra of silicon powder mounted on TBAF(tBuOH)_4_. (**a**-i) at room temperature, (**a**-ii) 100 °C, and (**a**-iii, **b**-i) after addition of EtOH vapor. The sample was then treated with CO_2_ two times (1st: **b**-ii, 2nd: **b**-iii).

The catalytic reaction pathway for the CO_2_ reductive transformation to amides was investigated. The time courses of amide and formic acid production were explored. Formic acid was produced in the initial stage of the reaction, and the amount of formamide produced increased as the reaction proceeded. This suggests that the conversion of CO_2_ to amide is a sequential reaction via formic acid. In situ diffuse reflectance infrared Fourier transform spectroscopy (DRIFTS) measurements were conducted to confirm the reaction mechanism, as shown in Fig. 4. The Si powder was mounted on TBAF(tBuOH)_4_^39^ at room temperature, and the temperature was then increased to 100 °C. Before heating, no clear signal was observed in the wavenumber range of approximately 1700–2100 cm^−1^ (Fig. 4a-i). In contrast, a broad signal appeared at approximately 1900 cm^−1^ with a broad shoulder at 2000–2100 cm^−1^, assigned to the stretching vibrations of F-H^40^ and Si-H species on silicon^32, 33, 40^, respectively, after heating at 100 °C which induced the mixing of Si powder and TBAF (Fig. 4a-ii). The signal intensities increased after the addition of EtOH vapor as a proton source (Fig. 4a-iii). These results indicate that the reaction between the Si powder and TBAF yielded Si-H species. The subsequent addition of CO_2_ eliminated the Si-H peak and the appearance of a new signal at approximately 1600–1700 cm^−1^ was observed, indicating the formation of a formyl group (Fig. 4b)^41^. In summary, the reaction between fluoride and Si powder affords Si-H species that react with CO_2_ to produce a formate species, possibly SiOC(O)H.

**Fig. 5:**
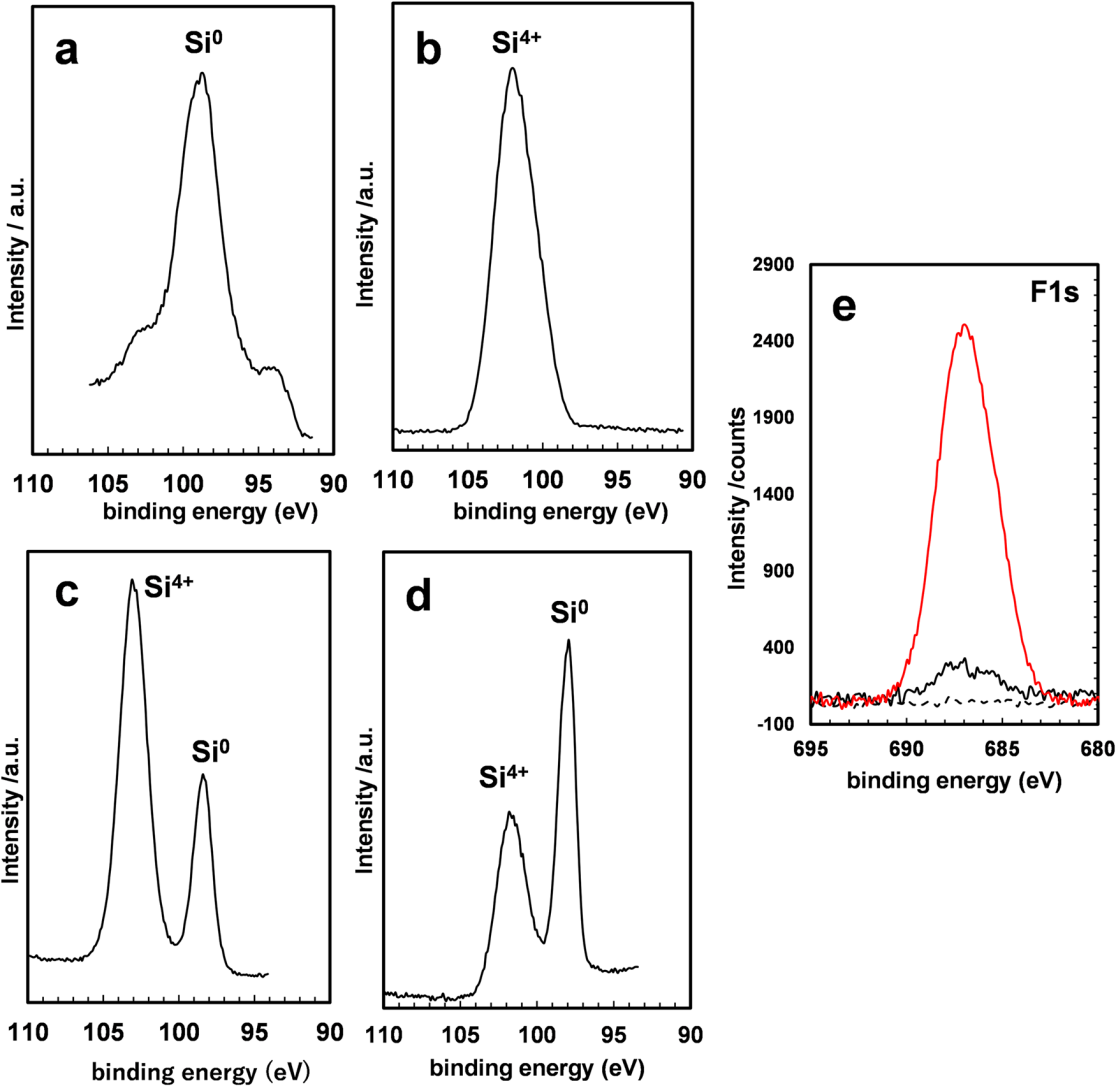
XPS spectra of fresh silicon powder and recovered solid. Si2p region of the (**a**) fresh silicon powder, and (**b**–**d**) recovered solid after the catalysis, (**b**) with TBAF under CO_2_, (**c**) without TBAF under CO_2_, and (**d**) with TBAF under Ar. **e** XPS spectra of the F1s region of fresh silicon powder (black dotted line), recovered solid after catalysis (black solid line), and the solid after milling (red solid line).

**Fig. 6:**
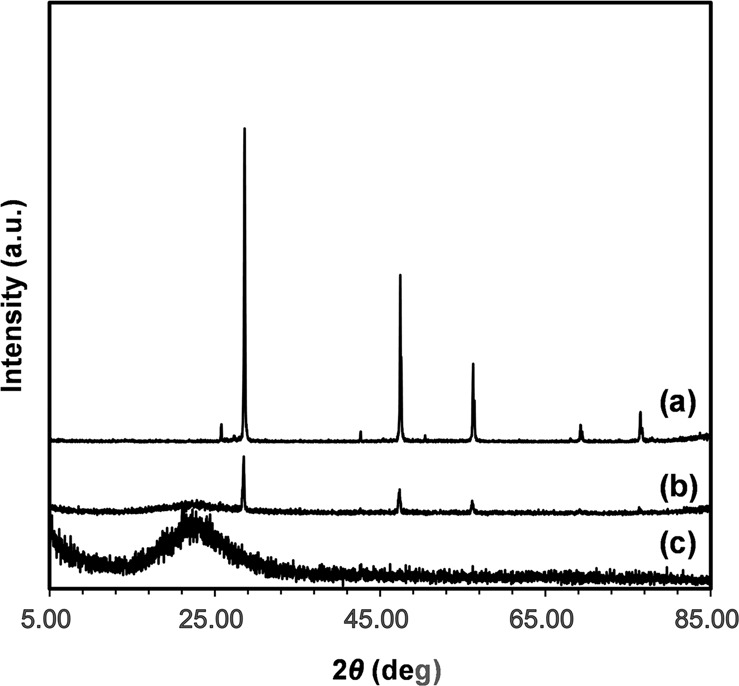
XRD patterns of fresh silicon powder and recovered solid. **a** Fresh silicon powder, and (**b**, **c**) recovered solid samples after the catalytic reaction with TBAF (**b**: 0.05 mmol, **c**: 1.0 mmol).

To confirm the detailed mechanism of the reaction between the solid Si powder and fluoride ions, X-ray photoelectron spectroscopy (XPS) measurements were conducted on the fresh Si powder and recovered solid after catalysis under these conditions. As shown in Fig. 5, a signal at 99 eV assigned to Si(0) was detected in the Si2p region of the fresh Si powder (Fig. 5a), and only the Si(+4) peak was detected in the recovered solid samples after catalysis in the presence of TBAF (Fig. 5b). This Si(+4) signal was observed, and no peak assigned to Si(0) was detected after milling the recovered sample, indicating that the oxidation occurred inside the Si powder. This result is also supported by XRD measurements of the recovered Si powder, as shown in Fig. 6. Before the reaction, the intensity of the XRD peaks derived from metallic silicon was high, suggesting that it existed as crystals of metallic silicon (Fig. 6(a)). In contrast, after the reaction, the intensity of the XRD peaks were reduced to 1/10 or less, and a broad peak was observed at approximately 20°, assigned to amorphous silica (Fig. 6(b)). These results indicate that metallic silicon was oxidized to SiO_2_ during the catalytic reaction, and the crystalline structure was degraded. In the sample with a larger amount of TBAF, the silicon metal-derived peaks disappeared completely, and the intensity of the amorphous silica-derived broad peak increased (Fig. 6(c)). Scanning electron microscopy–energy dispersive X-ray spectroscopy (SEM–EDS) analysis of the recovered solid also revealed the presence of a large amount of oxygen atoms compared with fresh Si powder (**see** Supplementary Figure S3)^34^.

Figure 5c shows the XPS spectrum of the recovered sample after the reaction with CO_2_ without TBAF. Interestingly, after milling of the recovered sample, the Si(0) peak was clearly detectable. This result suggests that fluoride ions enhance the oxidation of Si(0) inside the Si powder. The presence of CO_2_ is also necessary for the oxidation reaction of Si powder (Fig. 5d), indicating the reaction between Si(0) and CO_2_ in the presence of TBAF. In the XPS F1s region, a signal associated with the Si-F bond was detected at 685.4 eV after the catalytic reaction, as shown in Fig. 5e^42^. The signal intensity was significantly enhanced by milling the recovered solids. These results also support the interaction between the fluoride catalyst and the surface as well as the interior of the Si powder. Thus, TBAF degraded the crystalline structure of the Si powder, and the interior of the powder could be used for the reduction reaction.

**Fig. 7:**
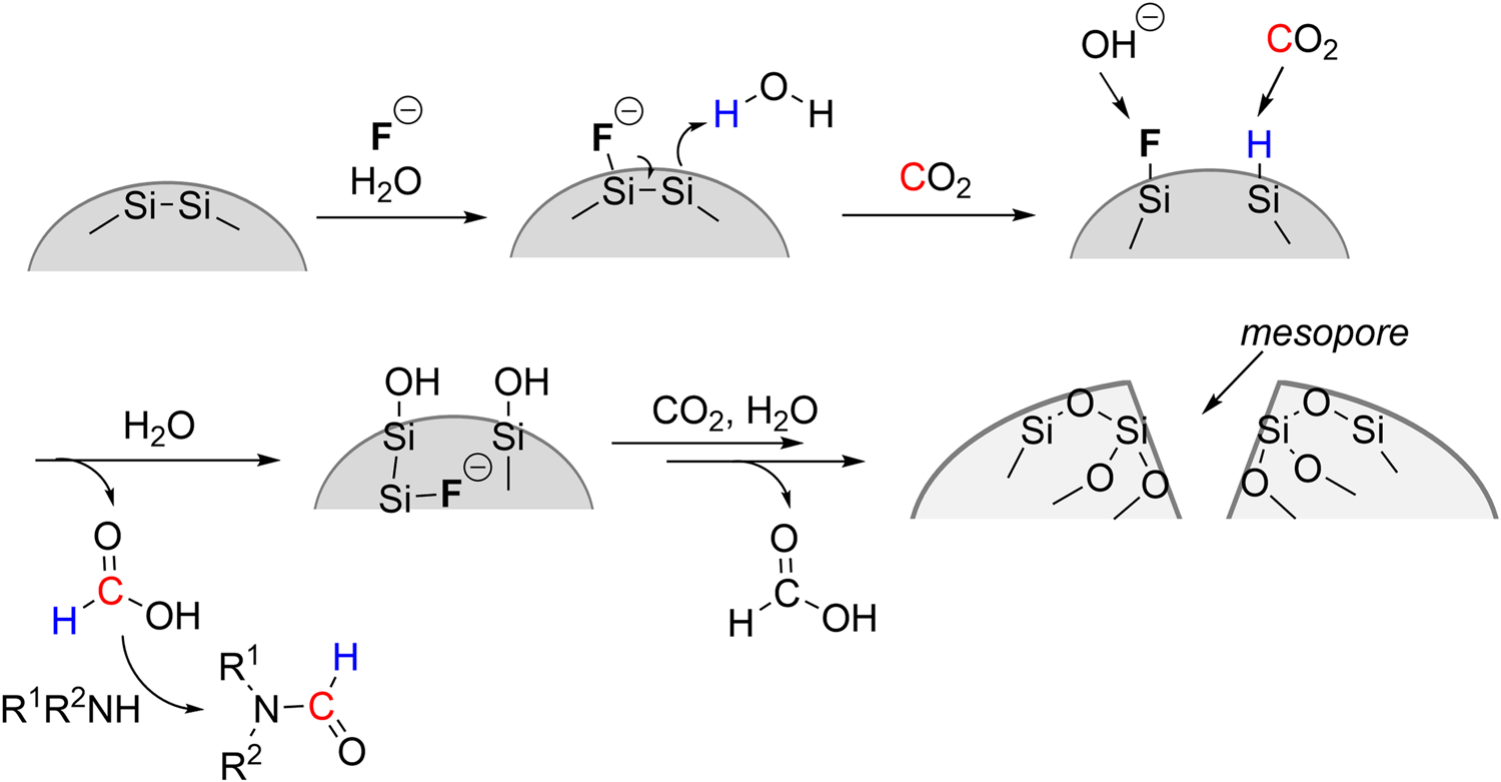
Schematic image of proposed reaction mechanism. Fluoride-catalyzed reduction of CO_2_ occrs at surface and inside bluk of silicon particle.

Based on a study that used disilane (R_3_Si-SiR_3_) as a reductant^43^, it is proposed that the coordination of fluoride ions to silicon results in the formation of Si-H and Si-F bonds by the nucleophilic attack of F to Si atoms with water molecules. The formation of hydrosilane and fluorosilane from disilane was also previously confirmed by a stoichiometric reaction^39^. Aprotic polar solvents, such as DMSO and NMP, have been reported to be effective for the hydrosilylation of CO_2_ by the coordination of the solvent to the Si atom^12^. Similarly, in this study, the reactivity of Si-H was also enhanced in the case using metallic silicon as a reducing agent in the presence of these solvents (Table 3). In situ FTIR analysis clearly revealed that the Si-H intermediate reacted with CO_2_ to afford the formate product (Fig. 4). The proposed reaction mechanism is shown in Fig. 7. During the reaction between Si-Si bonds, fluoride ions, and H_2_O, active Si-H species form, which reduce CO_2_ to formate. Regeneration of an active fluoride ion may occur by the reaction of Si-F with OH^-^ ions, because the reaction between Ph_3_SiF and cesium hydroxide affords an active fluoride species^29, 43^.

**Fig. 8:**
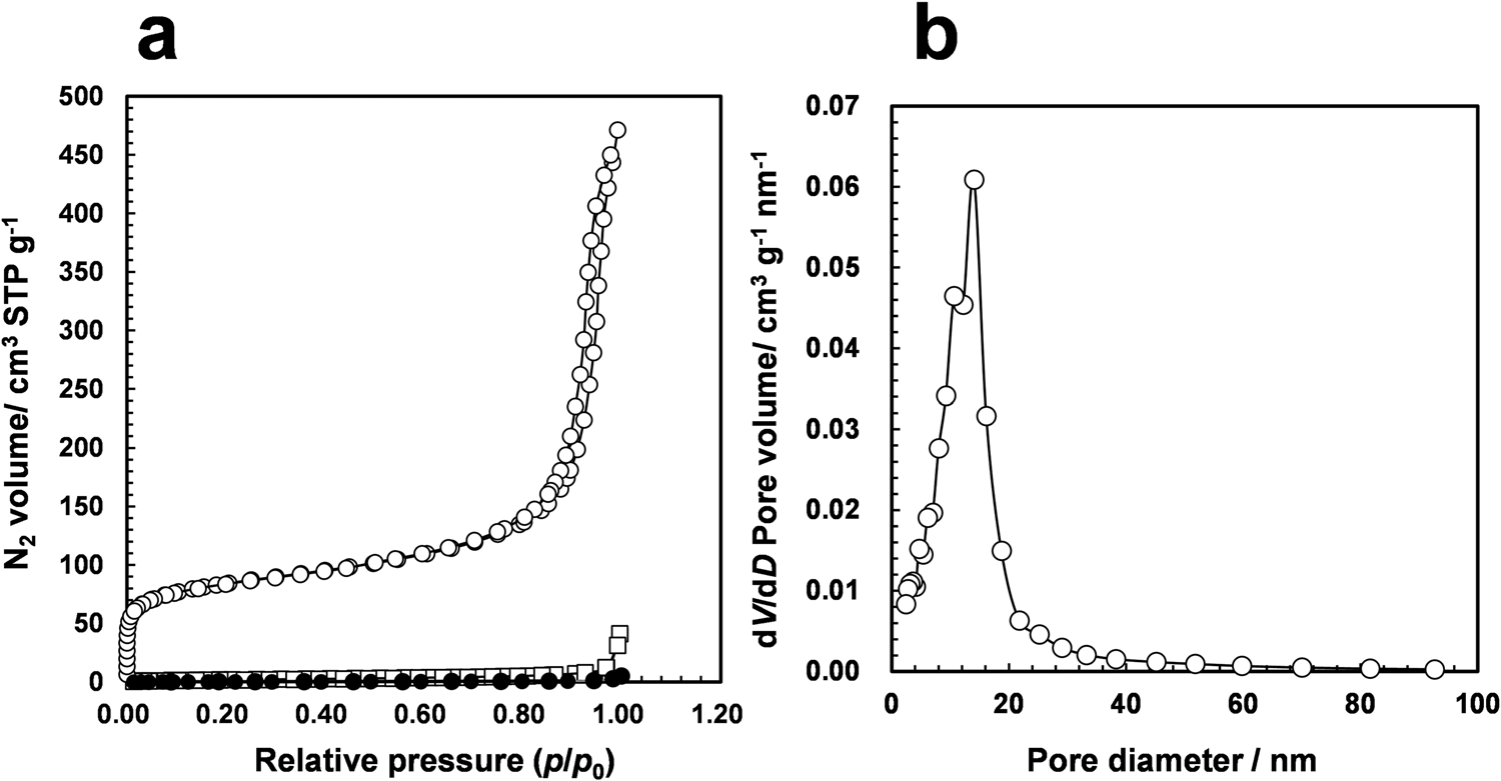
BET/BJH analysis resutls. **a** N_2_ adsorption-desorption isotherm of (open circle) recovered solid after fluoride catalysis, (open squre) fresh silicon powder, and (filled circle) recovered solid under reaction conditions without fluoride catalyst. **b** BJH pore size distribution of recovered sold after the catalysis.

XPS analysis revealed the oxidation of Si atoms occurred on the external and internal surfaces of the Si powder in the presence of the fluoride catalyst. Moreover, Si-F species were mainly detected in the interior of the particle. These results suggest that the fluoride ion connected to the external surface of the Si atom may diffuse to the interior of the Si particle, and the Si-H species generated in the interior of the Si particle also reduces CO_2_. Surprisingly, the surface area of the recovered solid after the reaction increased significantly (299.6 m^2^ g^−1^) compared with that of the fresh Si powder (8.2 m^2^ g^−1^) (Fig. 8a). Notably, without the fluoride catalyst, the surface area did not increase (1.2 m^2^ g^−1^, Fig. 8a). As shown in Fig. 8b, the Barrett–Joyner–Halenda (BJH) analysis of the spent Si powder used in the fluoride-catalyzed formamide synthesis contained mesopores with a diameter of 14 nm. These results indicate that the fluoride-catalyzed reaction occurred with the degradation of bulk silicon to form mesopores (Fig. 7). This is a first report catalysis of fluoride for reduction of CO_2_ using both surface and bulk silicon. After the formation of formic acid, the subsequent reaction with an amine affords formamide as the final product, which was revealed by time-course analysis.

## Conclusion

Silicon powder recovered from the solar panel production process was demonstrated to be an efficient reducing agent for CO_2_ in the synthesis of formamide. In the presence of a catalytic amount of TBAF, various amines reacted with CO_2_ to afford the corresponding target products in high yields. This is the first report of the catalytic conversion of CO_2_ to amides using metallic silicon as a reducing agent. XPS, FTIR, SEM–EDS, XRD, and N_2_ adsorption/desorption analyses indicated that both the external and internal surfaces of the Si particles were oxidized for the reduction of CO_2_ to formic acid via the formation of Si-H species. These unique effects of fluoride catalysts on Si may expand the possibility of various other reductive reactions, including tough/complex substrates such as CO_2_ and biomass, which will facilitate the efficient utilization of abundant resources.

## Methods

A silicon wafer used for solar panel production, the Czochralski monocrystalline silicon wafer, was obtained from the Renewable Energy Research Center, Advanced Industrial Science and Technology (AIST, Japan). The silicon wafer was crushed in an alumina mortar and sieved using an automatic sieve of size 20 μm. The prepared silicon wafer powder was added to an autoclave, and a fluoride catalyst, water, and carbon dioxide were introduced, and the mixture was stirred in an oil bath at a predetermined temperature for a predetermined time. Mesitylene was added to the solution as an internal standard after the reaction and the reaction products were qualitatively and quantitatively determined using nuclear magnetic resonance (NMR), gas chromatography–mass spectrometry (GC–MS), and gas chromatography with flame-ionization detection (GC–FID).

XPS analyses were conducted on an ULVAC-PHI Quntera SXM equipped with a dual Mg/Al X-ray source and a hemispherical analyzer operating in the field analyzer transmission mode. Excess charges on the samples were neutralized. The analysis chamber was conditioned to be less than 10^−7^ Pa during measurement. Spectra were acquired in the O 1 s, C 1 s, F 1 s, and Si 2p regions. Samples were powdered and attached to a stainless-steel plate with a carbon double tape. The C 1 s peak at a binding energy (BE) of 285 eV was taken as an internal reference.

The ATR-FTIR measurements were performed on Shimadzu IRTracer-100 equipped with a liquid-nitrogen-cooled MCT detector and variable temperature single-reflection ATR accessory (PIKE Technologies). The Si powder was mounted on TBAF(tBuOH)_4_ solid at room temperature. After the IR measurement at r.t., then, the solid was heated at 100 °C. After several minutes, the sample was treated by EtOH vapor followed by CO_2_ at 100 °C with IR monitoring.

N_2_ adsorption–desorption isotherms at 77 K were measured using a BELSORP mini (MicrotracBEL) system. Samples were prepared for N_2_ adsorption measurements by outgassing at 473 K for 2 h under vacuum to a final pressure of 1 Pa. The BET surface areas were estimated over the relative pressure (P/P_0_) range of 0.30–0.70. The pore size distribution was obtained from the analysis of the isotherms by using the Barrett–Joyner–Halenda (BJH) method.

## Supplementary information

Motokura_PR File

Supplementary Information

## Data Availability

Supplementary methods, NMR and MS spectra of isotopic experiment, and SEM-EDS images can be found in the Supplementary Information. Requests for additional data can be sent to the corresponding author.
