# Supplementary material for: CO2 conversion to formamide using a fluoride catalyst and metallic silicon as a reducing agent
Source: Commun Chem. 2022 Nov 16;5:150. doi: 10.1038/s42004-022-00767-4 (PMC9814565; doi:10.1038/s42004-022-00767-4)
Supplement: Supplementary file 2 — Supplementary Information [file 42004_2022_767_MOESM2_ESM.pdf]

Supplementary Information

CO_2 conversion to formamide using a fluoride catalyst and metallic silicon as a reducing agent

Ruopeng Wang,[1] Kaiki Nakao,[1,2] Yuichi Manaka,[2,3] Ken Motokura*[1,2]

[1] *Department of Chemistry and Life Science, Yokohama National University, 79-5 Tokiwadai, Hodogaya-ku, Yokohama 240-8501, Japan*

[2] *Department of Chemical Science and Engineering, School of Materials and Chemical Technology, Tokyo Institute of Technology, 4259 Nagatsuta-cho, Midori-ku, Yokohama 226-8502, Japan*

[3] *Renewable Energy Research Center, National Institute of Advanced Industrial Science and Technology (AIST), 2-2-9 Machiikedai, Koriyama 963-0298, Japan*

Contents

1. Experimental Section S2
1.1 General Methods S2
1.2 Preparation of silicon powder as the reducing agent S3
1.4 Typical procedure for fluoride-catalyzed formamide synthesis with silicon powder, amine, and H_2O S3
Figure S1 ^{1}H and ^{13}C NMR of formamide from $^{13}CO_2$ S4
Figure S2 Mass spectra of formamide from D_2O S5
Figure S3 SEM-EDS images S6

Supplementary Methods

1 Experimental Section

1.1 General Methods

Czochralski monocrystalline silicon wafers (solar grade, Si: >99.9999%) rejected during solar panel production were gifted by National Institute of Advanced Industrial Science and Technology (AIST). Tetrabutylammonium fluoride trihydrate (TBAF-3H_2O, >99%) was purchased from Kanto Chemical, Co. Inc. without further purification. $^{13}CO_2$ (^{13}C, 99%, <1% ^{18}O) was purchased from Cambridge Isotope Laboratories. D_2O was purchased from Kanto Chemical, Co. Inc. $CDCl_3$ (>99.8%) was purchased from Kanto Chemical, Co. Inc. *N*-methylpyrrolidone (NMP, dehydrated, >99%), Dimethylsulfoxide (DMSO, dehydrated, >99%), and other dehydrated solvents were purchased from Kanto Chemical, Co. Inc. and used without further purification. Unless otherwise mentioned, all other materials were purchased from Tokyo Chemical Industry Co., Ltd. Kanto Chemical Co., Inc. and Aldrich Inc.

The liquid ^1H and ^{13}C NMR spectra were measured with $CDCl_3$ as the solvent using Bruker AVANCE 400 (operating frequency are 400 and 100 MHz for ^1H and ^{13}C NMR measurement, respectively) and Bruker AVANCE HD500 (operating frequency are 500 and 125 MHz for ^1H and ^{13}C NMR measurement, respectively). Shimadzu QP2010 SE gas chromatograph-mass spectrometer (GC-MS) equipped with a DB-1 column and Shimadzu GC2025 gas chromatograph with flame ionization detection (GC-FID) were used for product characterization. Initially, the temperature was hold at 50°C for 6 minutes, then raised with a rate of 10°C/min to 280°C, then the temperature was hold at 280°C for the final 10 minutes. XPS analyses were conducted on an ULVAC-PHI Quntera SXM equipped with a dual Mg/Al X-ray source and a hemispherical analyzer operating in the field analyzer transmission mode. Excess charges on the samples were neutralized. The analysis chamber was conditioned to be less than 10^{-7} Pa during measurement. Spectra were acquired in the O 1s, C 1s, F 1s, and Si 2p regions. Samples were powdered and attached to a stainless-steel plate with a carbon double tape. The C 1*s* peak at a binding energy (BE) of 285 eV was taken as an internal reference. Powder X-ray diffraction (XRD) patterns were recorded using a Rigaku SmartLab diffractometer with Cu Kα radiation. SEM-EDS analysis of powdered silicon samples was performed by HITACHI SU8010. The ATR-FTIR measurements were performed on Shimadzu IRTracer-100 equipped with a liquid-nitrogen-cooled MCT detector and variable temperature single-reflection ATR accessory (PIKE Technologies). N_2 adsorption–desorption isotherms at 77 K were measured using a BELSORP mini (MicrotracBEL) system. Samples were prepared for N_2 adsorption measurements by outgassing at 473 K for 2 h under vacuum to a final pressure of 1 Pa. The BET surface areas were estimated over the relative pressure (P/P_0) range of 0.30–0.70. The pore size distribution was obtained from the analysis of the isotherms by using the Barrett–Joyner–Halenda (BJH) method.

1.2 Preparation of silicon powder as the reducing agent

Silicon wafers were crushed with alumina mortar to powdered form and sifted by automatic sieve with 300, 90, 40, and 20 μm mesh sizes. The different sizes of silicon powders were stored in containers with Argon atmosphere and moisture was kept low by storing the container inside a desiccator.

1.3 Typical procedure for fluoride-catalyzed formamide synthesis with silicon powder, amine, and H_2O

In a vial, TBAF-3H_2O (0.05 mmol), NMP (4 mL), morpholine (3.0 mmol), and a definite amount of deionized water (10 mmol) were mixed and introduced to a SUS autoclave. To a SUS autoclave, powdered silicon wafer (diameter < 20 μm, 5.0 mmol) was added. Afterwards, CO_2 was introduced and pressurized to 0.9MPa. The resulting mixture was stirred vigorously at 90 °C for 24 h. the reaction products were confirmed through GC-MS and ^{1}H NMR spectrometry. The formamide yield was determined by the liquid ^{1}H NMR in $CDCl_3$ solvent and/or GC-FID using the internal standard technique with 1,3,5-triisopropylbenzene as the internal standard. The formamide product was identified by their authentic samples and/or reported ^{1}H, ^{13}C NMR and MS data.

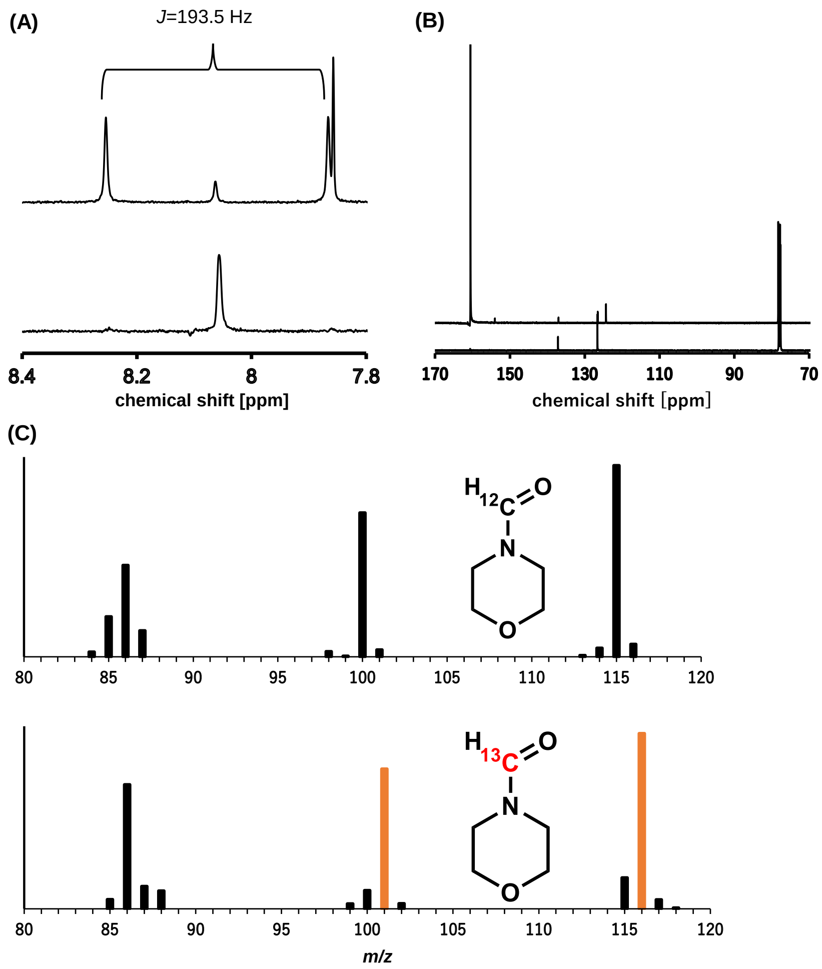

Figure S1. (A) ^{1}H NMR, (B) ^{13}C NMR, and (C) Mass spectra of the formamide product obtained from the reaction using $^{13}CO_2$. Reaction conditions: powdered silicon wafer (5 mmol), morpholine (1.0 mmol), $^{13}CO_2$ (0.1 MPa, balloon), H_2O (5 mmol), TBAF-3H_2O (0.05 mmol), DMSO (4 mL), 120 °C, 72 h.

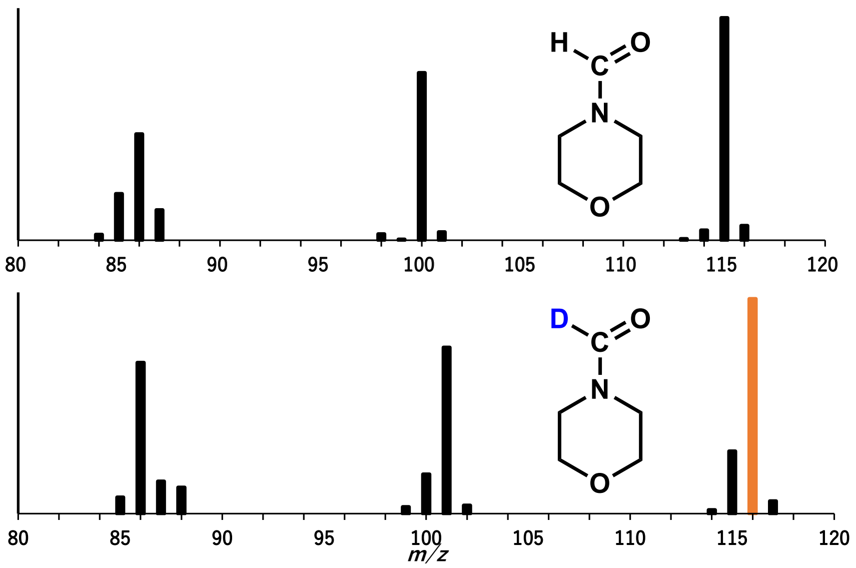

Figure S2. Mass spectra of the formamide product obtained from the reaction using H_2O and D_2O. Reaction conditions: powdered silicon wafer (5 mmol), morpholine (1.0 mmol), CO_2 (0.9 MPa), D_2O (10 mmol), TBAF-3H_2O (0.05 mmol), DMSO (4 mL), 120 °C, 72 h.

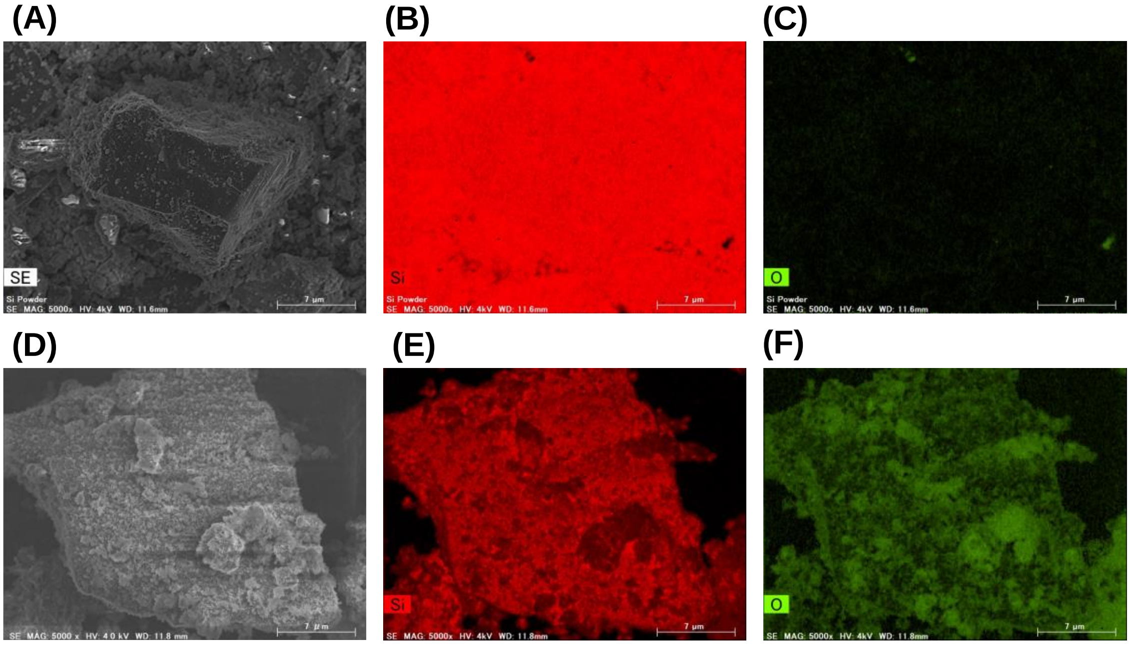

Figure S3 SEM image and EDS mapping (Si & O) of (A-C) fresh Si powder and (E-F) recovered solid after the catalysis.
