# Supplementary material for: CO2 conversion to formamide using a fluoride catalyst and metallic silicon as a reducing agent
Source: Commun Chem. 2022 Nov 16;5:150. doi: 10.1038/s42004-022-00767-4 (PMC9814565; doi:10.1038/s42004-022-00767-4)
Supplement: Supplementary file 1 — Motokura_PR File [file 42004_2022_767_MOESM1_ESM.pdf]

nature portfolio

Peer Review File

CO2 conversion to formamide using a fluoride catalyst and metallic silicon as a reducing agent

Open Access This file is licensed under a Creative Commons Attribution 4.0 International License, which permits use, sharing, adaptation, distribution and reproduction in any medium or format, as long as you give appropriate credit to the original author(s) and the source, provide a link to the Creative Commons license, and indicate if changes were made. In the cases where the authors are anonymous, such as is the case for the reports of anonymous peer reviewers, author attribution should be to 'Anonymous Referee' followed by a clear attribution to the source work. The images or other third party material in this file are included in the article's Creative Commons license, unless indicated otherwise in a credit line to the material. If material is not included in the article's Creative Commons license and your intended use is not permitted by statutory regulation or exceeds the permitted use, you will need to obtain permission directly from the copyright holder. To view a copy of this license, visit http://creativecommons.org/licenses/by/4.0/.

Reviewers' comments:

Reviewer #1 (Remarks to the Author):

This manuscript reports the formation of amides from CO_2 and various amines using metallic silicon as reducing agent in the presence of a catalytic amount of TBAF. The results revealed that the fluoride catalyst effectively oxidized Si atoms on the both surface and interior of the powdered silicon particles for the converion of CO_2 via an Si-H intermediate species. This study presents the first "shovel-like" catalysis of fluoride for the efficient reductive functionalization of CO_2. The mechanistic investigation is detailed discussed and I recommend it for publication based on following comments.
1."Shovel-like" fluoride catalysis in the maintext did not give any explanation on the "Shovel-like"or how to get "Shovel-like". The "Shovel-like" is only shown in the introduction part.
2.Metallic silicon recycling process was easily introduced. Scheme 1 was not important in the manuscript and can be removed.
3.Since F, Cl, Br, and I are the same maingroup, CO_2 conversion to formamide catalyzed by the organic salts (TBAF, TBACl…) should have a gradual order. However, why did only TBAF can catalyze CO_2 conversion to formamide? It is should be discussed in detail.
4.Table 2 summarized the results of the catalytic reactions under various reaction conditions. The authors should provide more insights into the results, for example, why did entry 2 maintain a high yield and the product was not obtained without H_2O or CO_2?
5.Table 2 showed an optimized formylated product was obtained in 94% yield at 6 atm, 120 °C, and 72 h. Why did authors select a completely conditions in Table 3 (CO_2, 9 atm, 90 °C, 24 h)? These results are confused in both Tables.
6.The yields of amide are similar using DMA, DMF, and NMP as solvent in Table 3, why did the authors used NMP as solvent in Table 4? Moreover, reaction conditions are also changed and it feels the authors selected quite arbitrary reaction conditions.
7.CO_2 conversion to formamide was performed on 120 °C (Tables 2 and 4) and 90 °C. For DRIFTS, why did the authors use 100 °C as measured temperature?
8.Figure 4b, pore diameters of three silicons should be provided and compared to discuss the change of structure of the silicons under the effect of TBAF.
9.The experimental section need provide some details, especially on the measurement of DRIFTS.
10.Very recent refs on CO_2 conversion should be cited see Chem Eng J DOI: 10.1016/j.cej.2020.12746, DOI: 10.1016/j.cej.2022.134956, and J. Mol. Liq. DOI: 10.1016/j.molliq.2021.118036.
11.Some minor errors should be corrected, for example, Page 5, "When 0.05 mmol of the TBAF catalyst was used..." couldn't be understood; Table 2, notes "DMSO (4 mL)" should be deleted; "In situ Fourier transform infrared" and "diffuse reflectance infrared Fourier transform spectroscopy" should only choose one term; and the authors should double check throughout.

Reviewer #2 (Remarks to the Author):

Sure, utilizing waste Si from the production process of solar panels to reduce CO_2 to organic chemicals and energetic compounds generates a circular economy that is beneficial to the environment. This paper discloses metallic silicon recovery from solar panel production and utilization as a reducing agent for formamide synthesis from CO_2 and amines. Various amines were converted to their corresponding amides with CO_2 and H_2O in the presence of a catalytic amount of tetrabutylammonium fluoride. The idea could originate from the same group Ref. 26, formamide synthesis from CO_2 using metallic silicon as a reducing agent. There are interesting results given in this work and significant insight into the working mechanism. In particular, fluoride catalyst effectively oxidizes Si atoms on the both surface and interior of the powdered silicon particles for the reduction of CO_2 via an Si-H intermediate species. Therefore, in my opinion, this work warrant to get published after careful revisions.

the role of cation part, i.e. ammonium cation in Scheme 4 and role of water are recommended to be further clarified. What are reasons such an excess of Si powder required ?

polymethylhydrosiloxane, a cheap byproduct of the silicone industry, also worked well as a reductant for CO_2 reductive functionalization with amines, see, ACS Sustain. Chem. Eng., 2018, 6, 8130-8135. In particular, the Si-H formation could be crucial and is recommended to be monitored by spectroscopic techniques.

Is there any by-products, such as double formylation, methylation, for example, in the case of entry 8 in Table 4. Please note that 2e, 4e, 6e reduction, namely, the generation of formamide, aminal and methylamine respectively through hierarchical 2-, 4- and 6-electron reduction of CO_2 see,Angew. Chem. Int. Ed., 2017, 56, 7425-7429.

Additional discussion at the beginning of the Introduction on carbon capture and utilisation (CCU) (integration of CO_2 capture and subsequent conversion) to organic compounds can be traced back to 2011 (e.g. CO_2 capture and activation and its subsequent conversion, Energy Environ. Sci., 2011, 4, 3971), and recently-published account (see, Acc. Chem. Res., 2019, 52, 2892-2903, and references are therein) is recommended.

Reviewer #3 (Remarks to the Author):

Motokura and co-workers described the recovery of silicon powder recovered from the solar panel production process for the synthesis of formamide. In the presence of a catalytic amount of TBAF, various amines reacted with CO_2 to afford the corresponding target products in high yields. Both the external and internal surfaces of the Si particles were oxidized for the reduction of CO_2 to formic acid via the formation of Si-H species. This work is a very important contribution for chemistry communities. This work affords an emerging chemical process for sustainable catalysis. It can be improved in the following aspects:

1. Have you check the C/H conservation before/after reaction? How about the exactly selectivity of formic acid?
2. How to distinguish the external and internal surfaces of Si particles?
3. The physical properties of Si reactants and SiO_2 products should be further explained. Please check the surface functional groups and particle size distribution of Si particles. The exactly composition of SiO_2 should be further checked and described.
4. In Figure 4b, please indicate the pore volume is dV/dD data.
5. Please provide more direct evidences to support the proposed mechanism shown in Scheme 4.
6. The key to improve the selectivity of formic acid should be further explained.

Point-to-point replies to Reviewers

Reviewer #1:

This manuscript reports the formation of amides from CO2 and various amines using metallic silicon as reducing agent in the presence of a catalytic amount of TBAF. The results revealed that the fluoride catalyst effectively oxidized Si atoms on the both surface and interior of the powdered silicon particles for the converion of CO2 via an Si-H intermediate species. This study presents the first "shovel-like" catalysis of fluoride for the efficient reductive functionalization of CO2. The mechanistic investigation is detailed discussed and I recommend it for publication based on following comments.

Comment 1."Shovel-like" fluoride catalysis in the maintext did not give any explanation on the "Shovel-like"or how to get "Shovel-like". The "Shovel-like" is only shown in the introduction part.

Reply 1: Thank you for the comment. According to the suggestion, a sentence have been added to main text as follows:

" …the fluoride-catalyzed reaction occurred with the degradation of bulk silicon to form mesopores (**Scheme 4**). This is a first report "shovel-like" catalysis of fluoride for reduction of CO_2 using both surface and bulk silicon."

Comment 2. Metallic silicon recycling process was easily introduced. Scheme 1 was not important in the manuscript and can be removed.

Reply 2: Thank you for the comment. Scheme 1 simply represents a novel silicon recycling process. According to the comment, the main text regarding metallic silicon utilization was simplified.

Old: "The current economic value of these panels is low, despite the high purity of silicon wafers, which accounts for approximately 2–3% of their weight.[20,21] A considerable amount of waste silicon is produced during wafer production. Therefore, utilizing waste Si from the production process of solar panels to reduce CO_2 to organic chemicals and energetic compounds generates a circular economy that is beneficial to the environment."
New: "The current economic value of these panels is low, despite the high purity of silicon wafers.[27,28] Therefore, utilizing waste Si from the production process of solar panels to reduce CO_2 to organic chemicals and energetic compounds generates a circular economy that is beneficial to the environment."

Comment 3. Since F, Cl, Br, and I are the same maingroup, CO2 conversion to formamide catalyzed by the organic salts (TBAF, TBACl…) should have a gradual order. However, why did only TBAF can catalyze CO2 conversion to formamide? It is should be discussed in detail.

Reply 3: Thank you for the comments. According to the comments, the order of the organic salts in Table 1 has been corrected. The high activity of TBAF compared with other halide salts is due to the high affinity of Si and F. For example, bond dissociation energy of Si-F (e.g. Me_3Si-F: 158 kcal mol^{-1}) is much higher than that of Si-Cl (Me_3Si-Cl: 117 kcal mol^{-1}). As a result, fluoride ion effectively interacts with Si atom.

This explanation has been shown in main text as follows:

"This is thought to be owing to the fact that the Si-F bond (e.g. Me_3Si-F: 158 kcal mol^{-1}) [30] has a much higher dissociation energy than other bonds such as Si-Si and Si-Cl (e.g. Me_3Si-$SiMe_3$: 79.3 kcal mol^{-1}; Me_3Si-Cl: 117 kcal mol^{-1}) [30], and thereby fluoride facilitates the Si-Si bond cleavage."

Comment 4. Table 2 summarized the results of the catalytic reactions under various reaction conditions. The authors should provide more insights into the results, for example, why did entry 2 maintain a high yield and the product was not obtained without H2O or CO2?

Reply 4. Thank you for the comment. The high yield under relatively mild conditions is due to the high activity of TBAF. Since both H_2O and CO_2 are converted to formamide, as shown in the isotopic experiments (Scheme 3), no product was obtained in entries 5-7. According to the comments, explanations have been added to main text as follows:

"The CO_2 pressure and reaction temperature were decreased to 4 atm and 90 °C, respectively, while maintaining a high yield (Entry 2), indicating high catalytic performance of TBAF. The product was not obtained without H_2O or CO_2 (Entries 5 and 6). These results suggest that both H_2O and CO_2 are converted to formamide product possibly."

Comment 5. Table 2 showed an optimized formylated product was obtained in 94% yield at 6 atm, 120 °C, and 72 h. Why did authors select a completely conditions in Table 3 (CO2, 9 atm, 90 °C, 24 h)? These results are confused in both Tables.

Reply 5: Thank you for the comment. As shown in Table 3, the highest yield (>99%) has been obtained under the conditions (CO_2: 9 atm, 90 °C, 24 h). According to the comment, an explanation for the further optimization have been added to main text.

"Further optimization of reaction conditions enabled a quantitative yield of the corresponding formamide product (>99%)."

Comment 6. The yields of amide are similar using DMA, DMF, and NMP as solvent in Table 3, why did the authors used NMP as solvent in Table 4? Moreover, reaction conditions are also changed and it feels the authors selected quite arbitrary reaction conditions.

Reply 6: Thank you for the comments. In Table 4, reaction conditions in entry 1 (morpholine) is same as Table 3. In the case of other amines with lower reactivity, the reaction conditions were optimized (120 oC, 72 h). NMP was used as a solvent to avoid amide exchange reaction between formamide product and solvent, such as DMF and DMA, under the high temperature in Table 4.

Comment 7. CO2 conversion to formamide was performed on 120 °C (Tables 2 and 4) and 90 °C. For DRIFTS, why did the authors use 100 °C as measured temperature?

Reply 7: The catalytic reaction occurred in the range of 90-120 °C. Therefore, the FT-IR measurement at 100 °C should be acceptable.

Comment 8. Figure 4b, pore diameters of three silicons should be provided and compared to discuss the change of structure of the silicons under the effect of TBAF.

Reply 8: As shown in Figure 4 and main text, other two samples show very lower surface area (8.2 and 1.2 m^2g^{-1}) compared with the sample after the catalysis (299.6 m^2g^{-1}). BJH analysis also indicates that other two samples did not show porous structure.

Comment 9. The experimental section need provide some details, especially on the measurement of DRIFTS.

Reply 9: Thank you for the suggestion. Details for spectroscopic measurements have been added to the Experimental section, as follows.

“XPS analyses were conducted on an ULVAC-PHI Quntera SXM equipped with a dual Mg/Al X-ray source and a hemispherical analyzer operating in the field analyzer transmission mode. Excess charges on the samples were neutralized. The analysis chamber was conditioned to be less than 10^{-7} Pa during measurement. Spectra were acquired in the O 1s, C 1s, F 1s, and Si 2p regions. Samples were powdered and attached to a stainless-steel plate with a carbon double tape. The C 1*s* peak at a binding energy (BE) of 285 eV was taken as an internal reference.

The ATR-FTIR measurements were performed on Shimadzu IRTracer-100 equipped with a liquid-nitrogen-cooled MCT detector and variable temperature single-reflection ATR accessory (PIKE Technologies). The Si powder was mounted on TBAF(tBuOH)$_4$ solid at room temperature. After the IR measurement at r.t., then, the solid was heated at 100 °C. After several minutes, the sample was treated by EtOH vapor followed by CO_2 at 100 °C with IR monitoring.

N_2 adsorption–desorption isotherms at 77 K were measured using a BELSORP mini (MicrotracBEL) system. Samples were prepared for N_2 adsorption measurements by outgassing at 473 K for 2 h under vacuum to a final pressure of 1 Pa. The BET surface areas were estimated over the relative pressure (P/P_0) range of 0.30–0.70. The pore size distribution was obtained from the analysis of the isotherms by using the Barrett–Joyner–Halenda (BJH) method.”

Comment 10. Very recent refs on CO2 conversion should be cited see Chem Eng J DOI: 10.1016/j.cej.2020.12746, DOI: 10.1016/j.cej.2022.134956, and J. Mol. Liq. DOI: 10.1016/j.molliq.2021.118036.

Reply 10: Thank you for the comments. References, 10.1016/j.cej.2022.134956 and 10.1016/j.molliq.2021.118036 have been cited as ref [18] and [19].

Comment 11. Some minor errors should be corrected, for example, Page 5, “When 0.05 mmol of the TBAF catalyst was used...” couldn’t be understood; Table 2, notes “DMSO (4 mL)” should be deleted; “In situ Fourier transform infrared” and “diffuse reflectance infrared Fourier transform spectroscopy” should only choose one term; and the authors should double check throughout.

Reply 11: Thank you for the comments. These mistakes have been corrected.

Reviewer #2:
Sure, utilizing waste Si from the production process of solar panels to reduce CO2 to organic chemicals and energetic compounds generates a circular economy that is beneficial to the environment. This paper discloses metallic silicon recovery from solar panel production and utilization as a reducing agent for formamide synthesis from CO2 and amines. Various amines were converted to their corresponding amides with CO2 and H2O in the presence of a catalytic amount of tetrabutylammonium fluoride. The idea could originate from the same group Ref. 26, formamide synthesis from CO2 using metallic silicon as a reducing agent. There are interesting results given in this work and significant insight into the working mechanism. In particular, fluoride catalyst effectively oxidizes Si atoms on the both surface and interior of the powdered silicon particles for the reduction of CO2 via an Si-H intermediate species. Therefore, in my opinion, this work warrant to get published after careful revisions.

Comment 1: the role of cation part, i.e. ammonium cation in Scheme 4 and role of water are recommended to be further clarified. What are reasons such an excess of Si powder required ?

Reply 1: Role of cation: The effect of cation on the catalytic reaction is summarized in Table 1. Cation controls both fluoride reactivity and solubility of salt, resulting the catalytic performance is affected by cation. However, the cation does not affect the reaction mechanism directly, therefore, we did not show cation in Scheme 4.

Role of water: As shown in Scheme 4, water is a hydrogen source of formamide. As shown in Scheme 3, hydrogen atom of formyl group in the formamide product is originated from water.

Excess Si: Due to the solid nature of silicon powder, its reactivity is not high, therefore, addition of excess amount of silicon powder is necessary.

For example, these sentences in main text which explain the above questions have been presented / added :

"These results suggest that the activity of the catalyst is related to the counter cation size and solubility in fluoride salts."

"During the reaction between Si-Si bonds, fluoride ions, and H_2O, active Si-H species form, which reduce CO_2 to formate."

"The use of excess amount of powdered silicon enables effective production of the formamide."

Comment 2. polymethylhydrosiloxane, a cheap byproduct of the silicone industry, also worked well as a reductant for CO2 reductive functionalization with amines, see, ACS Sustain. Chem. Eng., 2018, 6, 8130-8135. In particular, the Si-H formation could be crucial and is recommended to be monitored by spectroscopic techniques.

Reply 2: Thank you for the comment. According to the comment, the work was cited as follows:

"The use of a cheap reducing agent, polymethylhydrosilane, was also investigated using an organocatalyst.[20]"

"[20] X.-Y. LI, S.-S. Zheng, X.-F. Liu, Z.-W. Yang, T.-T. Tan, A. Yu, L.-N. He, Waste Recycling: Ionic Liquid-Catalyzed 4-Electron Reduction of CO_2 with Amines and Polymethylhydrosiloxane Combining Experimental and Theoretical Study. *ACS Sustainable Chem. Eng.* **2018**, *6*, 8130-8135."

The formation of Si-H was monitored by in-situ FT-IR. The results are summarized in main text and Figure 1.

Comment 3. Is there any by-products, such as double formylation, methylation, for example, in the case of entry 8 in Table 4. Please note that 2e, 4e, 6e reduction, namely, the generation of formamide, aminal and methylamine respectively through hierarchical 2-, 4- and 6-electron reduction of CO2 see,Angew. Chem. Int. Ed., 2017, 56, 7425-7429.

Reply 3: Thank you for the comment. In our reaction system, very small amount (trace) of methyl amine was detected. Other reduction product was not detected.

According to the comment, the reference has been cited as [17].

[17] X.-F. Liu, X.-Y. Li, C. Qiao, H.-C. Fu, L.-N. He, Betaine Catalysis for Hierarchical Reduction of CO_2 with Amines and Hydrosilane to Form Formamides, Aminals, and Methylamines. *Angew. Chem. Int. Ed.* **2017**, *56*, 7425-7429.

Comment 4. Additional discussion at the beginning of the Introduction on carbon capture and utilisation (CCU) (integration of CO2 capture and subsequent conversion) to organic compounds can be traced back to 2011 (e.g. CO2 capture and activation and its subsequent conversion, Energy Environ. Sci., 2011, 4, 3971), and recently-published account (see, Acc. Chem. Res., 2019, 52, 2892-2903, and references are therein) is

recommended.

Reply 4: Thank you for the useful suggestion. These references have been cited in Introduction section.

"[21] Z.-Z. Yang, L.-N. He, Y.-N. Zhao, B. Li, B. Yu, CO_2 capture and activation by superbase/polyethylene glycol and its subsequent conversion. *Energy Environ. Sci.*, **2011**, *4*, 3971-3975.

[22] S. Kar, A. Goeppert, G. K. S. Prakash, Integrated CO_2 Capture and Conversion to Formate and Methanol: Connecting Two Threads. *Acc. Chem. Res.* **2019**, *52*, 2892−2903"

Reviewer #3:

Motokura and co-workers described the recovery of silicon powder recovered from the solar panel production process for the synthesis of formamide. In the presence of a catalytic amount of TBAF, various amines reacted with CO2 to afford the corresponding target products in high yields. Both the external and internal surfaces of the Si particles were oxidized for the reduction of CO2 to formic acid via the formation of Si-H species. This work is a very important contribution for chemistry communities. This work affords an emerging chemical process for sustainable catalysis. It can be improved in the following aspects:

Comment 1. Have you check the C/H conservation before/after reaction? How about the exactly selectivity of formic acid?

Reply 1: Thank you for the comment. Regarding C/H conversion and selectivity, we have used excess amount of CO_2 and H_2O (proton source). For example, the yield based on CO_2 used is calculated to be *ca.* 18%, in Table 1 (TBAF catalyst).

According to the comment, a sentence has been added to main text as follows:
"The yield based on CO_2 used is calculated to be *ca.* 18%."

Comment 2. How to distinguish the external and internal surfaces of Si particles?

Reply 2: Thank you for the comment. XPS analysis reveals surface composition (Si(+4)/Si(0)), while XRD analysis indicates the Si(0) crystal structure of whole Si particle. The presence of mesopores is detectable by N_2 adsorption-desorption analysis (Figure 4). After the catalytic reaction, Si particle shows porous structure. Not only XPS (Figure 2) but also XRD (Figure 3) analysis revealed that Si(0) composition was significantly decreased, indicating both external surface and internal surface of Si was oxidized.

Comment 3. The physical properties of Si reactants and SiO2 products should be further explained. Please check the surface functional groups and particle size distribution of Si particles. The exactly composition of SiO2 should be further checked and described.

Reply 3: Thank you for the comment. The reaction mechanism shown in Scheme 4, is strongly supported by N_2 adsorption-desorption (Figure 4, porous structure), and XRD

(Figure 3) and XPS (Figure 2) analysis (SiO_2 formation). In addition, the presence of Si-F bond into the internal surface is also supported by F 1s XPS (Figure 2). We think these results indicate the mechanism. On the other hand, particle size and surface functional groups are also interesting properties. We will try to analyze them as a future work with a systematically experiment.

Comment 4. In Figure 4b, please indicate the pore volume is dV/dD data.

Reply 4: Thank you for the comment. Figure 4B has been corrected.

Comment 5. Please provide more direct evidences to support the proposed mechanism shown in Scheme 4.

Reply 5: Proposed mechanism is supported by analysis techniques. For example, porous structure of recovered solid is revealed by N_2 adsorption-desorption (Figure 4). The surface / bulk composition of the sold is indicated by XRD (Figure 3) and XPS (Figure 2) analysis. The presence of Si-F bond during the reaction is also supported by F 1s XPS (Figure 2). In addition, in-situ FT-IR analysis indicates that the formation of an active Si-H during the reaction (Figure 1). We think that these results are direct evidences for the reaction mechanism shown in Scheme 4.

Comment 6. The key to improve the selectivity of formic acid should be further explained.

Reply 6: As shown in Scheme 4, the formic acid intermediate is smoothly reacted with an amine to give formamide as a final product. After the complete conversion of the amine, formic acid formed selectively.

This manuscript focuses on the production of the formamide in high yield and selectivity (Table 4).

REVIEWERS' COMMENTS:

Reviewer #1 (Remarks to the Author):

The authors carefully revised the manuscript following my suggestions. I would recommend Accept.

Reviewer #2 (Remarks to the Author):

In my opinion, all the queries and suggestions the reviewers raised have been handled with care. I am satisfied with sufficient changes. Therefore, I recommend acceptance of this work without any further changes.

Reviewer #3 (Remarks to the Author):

It can be accepted now.
